# Variations in Serum Albumin Levels Over Time in Patients Treated With Conventional Hemodialysis or Expanded Hemodialysis: A Cohort Study

**DOI:** 10.1111/hdi.13232

**Published:** 2025-04-29

**Authors:** Juan C. Castillo, Jasmin Vesga, Angela Rivera, Peter Rutherford, Ricardo Sanchez, Henry Oliveros, Bengt Lindholm, Mauricio Sanabria, the Colombian Registry of Expanded Hemodialysis Investigators, David Camargo, Anthony Martinez, Sylvia Quiñones, Daniel A. Ducuara, Mario Munevar, Edward Martinez, Maria P. Dazzarola, Rafael Gomez, Alvaro del Castillo

**Affiliations:** ^1^ Renal Care Services Soacha Bogota Colombia; ^2^ Renal Care Services Colombia Bucaramanga Colombia; ^3^ Baxter Healthcare Corporation Deerfield Illinois USA; ^4^ Baxter Healthcare Corporation Zurich Switzerland; ^5^ Clinical Research Institute, Faculty of Medicine National University of Colombia Bogota Colombia; ^6^ Faculty of Medicine Sabana University Bogota Colombia; ^7^ Renal Medicine and Baxter Novum Karolinska Institutet Stockholm Sweden; ^8^ Baxter Renal Care Services, Latin‐America Bogota Colombia

**Keywords:** HDx therapy, hemodialysis, high‐flux hemodialysis, serum albumin, Theranova dialyzer

## Abstract

**Introduction:**

Hypoalbuminemia is a well‐established risk factor for mortality in chronic hemodialysis (HD) patients. To evaluate the association of time‐varying serum albumin with the type of dialyzer, we analyzed serum albumin over time in two cohorts of HD patients, one receiving HDx therapy enabled by the Theranova dialyzer and the other conventional HD with high‐flux dialyzer (HF‐HD).

**Methods:**

In this cohort study, 1092 prevalent adult HD patients (mean age 61 years; 62% men; 42% had diabetes; 19% had cardiovascular disease) at Renal Care Services Colombia undergoing either HDx therapy enabled by Theranova dialyzer (*n* = 559) or HF‐HD (*n* = 533) were enrolled between September 1, 2017, and November 30, 2017, and then underwent repeated measurements of serum albumin for up to 48 months. Sociodemographic and clinical, and laboratory characteristics at baseline were recorded, and a repeated‐measures analysis of variance (ANOVA) was conducted to examine differences in means of serum albumin at different time points. To evaluate the association between dialysis membrane and albumin levels during the follow‐up, a linear panel regression analysis was performed, allowing control for imbalances in the cohorts of baseline clinical and demographic variables, as well as the time‐dependent variables.

**Results:**

Mean albumin concentration remained above 3.8 g/dL and did not differ over time between HDx and HF‐HD (*p* = 0.789). No association (*p* = 0.208) between serum albumin levels varying over time and the use of the Theranova dialyzer was found in the linear panel regression model. However, serum albumin was linked to both inflammatory and nutritional markers, including C‐reactive protein, ratio of platelets to lymphocytes, and protein‐energy wasting.

**Conclusion:**

Variations in serum albumin levels over time were associated with protein‐energy wasting, inflammation, high age, vascular access, and hospitalizations, but not with the type of dialyzer.

## 1 Introduction

Malnutrition and inflammation are common in patients on chronic dialysis and are associated with adverse clinical outcomes [1, 2]. Several factors are interrelated within these two conditions, configuring a complex syndrome that can be expressed clinically in various ways, one of them being hypoalbuminemia [3, 4]. The term protein‐energy wasting (PEW) has been coined to denote a loss of body protein mass and fuel reserves in patients with chronic kidney disease [5]. In the definition of PEW, hypoalbuminemia is a factor that is neither necessary nor sufficient, even though it is frequently present in this condition and is known as a strong predictor of negative outcomes [5].

Albumin is a negative acute phase protein, whose serum levels decrease in acute inflammatory events [3]. The hypoalbuminemia of patients on dialysis can be explained by a decrease in hepatic synthesis due to inflammatory processes that occur in patients with kidney failure or reflect malnutrition because of a decrease in protein‐calorie intake, or be secondary to albumin loss through the dialyzer [4]. The association of time‐varying hypoalbuminemia with total and cardiovascular mortality was reported to differ from that of basal fixed single measurements of serum albumin or other markers of malnutrition or inflammation [6].

An important moment in the technological journey of chronic hemodialysis came with the development of medium cut‐off (MCO) membranes, which expanded the possibilities of clearing medium molecules. Thanks to their design with a cut‐off point very close to or lower than that of albumin, the use of MCO membranes resulted in improved removal of uremic solutes, but without significant leakage of albumin or with only marginal losses [7]. A study comparing HDx therapy enabled by Theranova dialyzer versus eight different dialyzers used in online hemodiafiltration (OL‐HDF) found that the removal of middle‐sized molecules was very similar, without relevant changes in albumin loss [8]. Likewise, a pilot randomized controlled trial of HDx versus HF‐HD and OL‐HDF showed that HDx therapy removed middle molecules more effectively than HF‐HD and even exceeded the performance of OL‐HDF; albumin loss was moderate with the MCO membrane, but greater than with HF‐HD and OL‐HDF [9].

A large real‐world cohort study in Colombia with 992 patients receiving HDx therapy enabled by the Theranova dialyzer and followed for 1 year showed only a slight decrease in albumin levels of −1.2% from baseline levels, without the presence of adverse events related to the use of the MCO membrane in 130,601 sessions [10]. Added to the previous evidence, a cohort study of 1092 patients followed for 2 years and dialyzed with the MCO membrane versus HF membranes showed that there was no difference between the two groups in albumin levels according to the type of dialyzer used [11]. However, doubt remains regarding the possible impact that the discrete increase in albumin losses through HDx therapy enabled by the MCO membrane may have on outcomes such as long‐term mortality [12].

The objective of the present study is to estimate the association between the use of HDx therapy enabled by MCO membranes and HF membranes and trajectories of repeated measurements of albumin and other variables of interest over a 4‐year follow‐up period.

## 2 Methods

### 2.1 Study Design and Patients

This is a retrospective, observational, multicenter, cohort study of prevalent patients undergoing HD (defined as having received HD for 90 days or more) receiving either HDx therapy by Theranova dialyzer (*n* = 559) or conventional HD using high flux membrane (*n* = 533). Patients were enrolled in the study from September 1, 2017, to November 30, 2017, and received their dialysis treatment at clinical centers belonging to the Renal Care Services network in Colombia with a maximum follow‐up of 4 years. The inclusion criteria were age greater than 18 years, preceding HD treatment > 90 days, treatment either by HDx therapy enabled by MCO membrane or conventional HD using an HF membrane for a minimum of 4 h, 3 times per week. The exclusion criteria were a life expectancy of less than 6 months, active infection, metastatic disease, or a Charlson comorbidity index score > 8. There was no specific clinical indication for the use of HDx therapy or HF membranes at the time of inception; this depended on the availability of the membranes at the clinical sites and assignment to one or the other of the two membranes was at the discretion of the treating nephrologists. Censored events were a kidney transplant, loss of follow‐up, suspension of dialysis therapy, change of dialysis provider, change of dialysis modality, change of type of membrane, and recovered kidney function.

## 3 Data Source and Analysis

### 3.1 Baseline Characteristics of the Patients

Demographic and clinical baseline variables included age, sex, ethnicity, dialysis vintage, Charlson comorbidity index score, Karnofsky scale score, and PEW. PEW was measured by applying the four diagnostic criteria: (1) altered serum biochemistry indicated by a serum albumin level of < 3.8 g/L or total cholesterol < 100 mg/dL; (2) decreased body mass status identified by a body mass index (BMI) of < 23 kg/m^2^ or < 10% total body fat; (3) muscle wasting defined by the lean tissue index; and (4) low dietary protein intake determined by the normalized protein equivalent of a total nitrogen appearance of < 0.8 g/kg/day, history of cardiovascular disease or diabetes, serum levels of hemoglobin, phosphorus, albumin (bromocresol green), potassium, platelet‐lymphocyte ratio, and high sensitive C‐reactive protein (hs‐CRP), and data on Kt/V single pool, and urine output in milliliters per day. Additionally, we collected data regarding vascular access, dialysis flow rate, ultrafiltration, and blood flow rate. All data were retrospectively collected from the system for electronic medical records of Renal Care Services called Versia. An internal audit was carried out as part of a data quality assurance process.

### 3.2 Study Outcomes

The primary objective was to determine the trend in serum albumin levels throughout follow‐up and its relationship to the type of dialysis membrane. Secondary outcomes included associations of serum albumin with other factors.

### 3.3 Statistical Analysis

Data are presented as mean and standard deviation (SD) for variables with normal distribution and as median and interquartile range for variables with non‐normal distribution. Categorical variables are presented as frequencies and percentages. Missing values were imputed using the last observation carried forward (LOCF) method; given the availability in some cases of additional measurements to those scheduled every 6 months, this strategy can take advantage of nearby values related to closer measurements. The percentage of missing data was 14% for hs‐CRP, 11% for PEW, 5.7% for platelet–lymphocyte ratio, and 2.1% for albumin. A repeated‐measures analysis of variance (ANOVA) was conducted to examine the differences in means of serum albumin, inflammation (hs‐CRP), nutrition (PEW score), and dialysis efficacy (KT/V urea) biomarkers at different time points. Additionally, the percentage change from the previous measurement was compared between the two cohorts using Pearson's *χ* ^2^ test. To evaluate the association between dialysis membrane and albumin levels during the follow‐up, controlling for confounding variables, a linear panel regression analysis was performed. The panel structure consisted of 1092 patients in whom outcome (albumin levels) and exposure (type of membrane) were measured repeatedly every 6 months. The following variables were included as control variables: C‐reactive protein (mg/L), ratio platelets/lymphocytes, PEW, age, history of diabetes, history of major cardiovascular events, urinary output ≥ 150 mL/day, vascular access, dialysis vintage, and number of hospitalizations. To incorporate the effect of baseline variables that did not change over time (for estimating effects of time‐constant regressors), a random effects model was used. Linear panel regression models help address variability both from fixed effects, which correspond to the factors of interest, and from random effects, which account for unobserved individual differences among research subjects. This approach is particularly valuable in studies with repeated measures, as it allows for modeling the intrinsic correlation of observations within the same subject, thus controlling for potential confounding due to unexplained individual variability. By considering both between‐subject variability and potential confounders, the mixed linear regression model enhances the precision of estimates, reducing bias and increasing the validity of the results. Multicollinearity was evaluated by calculating variance inflation factor (VIF) values. A robust standard error estimation method was employed using an extension of the Huber–White sandwich estimator. Stata 16 (StataCorp. 2019. Stata statistical software: Release 16. College Station, TX: StataCorp LLC.) was used for statistical analyses.

## 4 Results

**FIGURE 1:**
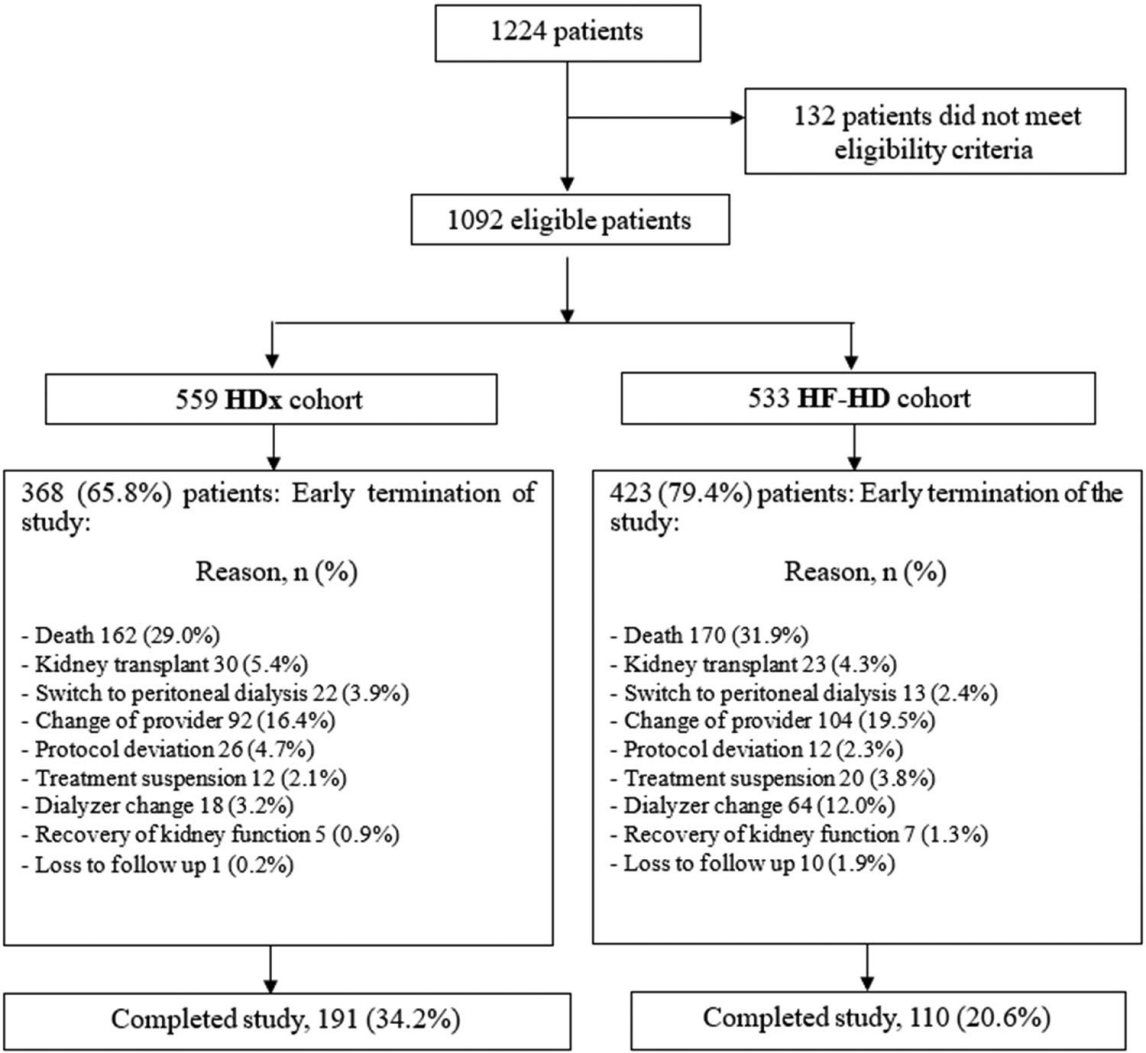
Flowchart of the recruitment of patients undergoing HDx therapy enabled by Theranova dialyzer or high‐flux hemodialysis (HF‐HD) into the study.

A total of 1092 patients met the participation criteria and were included in the two arms of the study: 533 patients in the HF‐HD cohort versus 559 patients in the HDx cohort (Figure 1). The majority (62%) were men, the mean age was 60.6 years, 42% had a history of diabetes, 19% had cardiovascular disease, and 15% had PEW (Table 1). Regarding the characteristics of hemodialysis prescription, the blood pump flow was, on average, 340 mL/h, and the dialysate flow was 484 mL/h, and the percentage of patients with arteriovenous fistula as vascular access was 82%.

**TABLE 1:** Baseline demographic and clinical characteristics.

Characteristics	HF‐HD (*N* = 533)	HDx (*N* = 559)	Full sample (*N* = 1092)	*p*
Age (years)	60.38 (14.87)	60.84 (14.93)	60.62 (14.90)	0.610
Sex				
Male	347 (65.10%)	335 (59.93%)	682 (62.45%)	0.078
Female	186 (34.90%)	224 (40.07%)	410 (37.55%)	
Ethnicity				
Black	55 (10.32%)	28 (5.01%)	83 (7.60%)	< 0.001
Mixed ancestry	478 (89.68%)	531 (94.99%)	1009 (92.40%)	
History of diabetes	214 (40.15%)	245 (43.83%)	459 (42.03%)	0.220
Cardiovascular disease	86 (16.14%)	120 (21.47%)	206 (18.86%)	0.024
Karnofsky scale	75.61 (15.91)	78.53 (13.87)	77.11 (14.97)	0.001
Charlson comorbidity index	2.04 (1.79)	2.18 (1.95)	2.11 (1.88)	0.240
Urinary output (mL/day)				
< 150	391 (73.36%)	409 (73.17%)	800 (73.26%)	0.940
≥ 150	142 (26.64%)	150 (26.83%)	292 (26.74%)	
Serum albumin (g/dL)	3.95 (0.45)	4.02 (0.34)	3.99 (0.40)	0.005
Serum hemoglobin (g/dL)	11.56 (1.91)	11.88 (1.74)	11.72 (1.83)	0.004
Serum calcium (mg/dL)	8.79 (0.74)	8.94 (0.82)	8.87 (0.78)	0.002
Serum phosphorus (mg/dL)	4.57 (1.54)	4.63 (1.40)	4.60 (1.47)	0.460
Serum potassium (mEq/L)	5.17 (0.80)	5.23 (0.78)	5.20 (0.79)	0.220
C‐reactive protein (mg/L)	3.90 (6.58)	1.24 (3.29)	2.30 (5.05)	< 0.001
KT/V single pool	1.59 (0.33)	1.66 (0.36)	1.62 (0.35)	< 0.001
Ratio platelets/lymphocytes	155.84 (79.39)	138.90 (70.84)	146.77 (75.37)	< 0.001
Protein energy wasting (PEW)				
No	459 (86.12%)	464 (83.01%)	923 (84.52%)	0.160
Yes	74 (13.88%)	95 (16.99%)	169 (15.48%)	
Dialysate flow (mL/min)	480.07 (47.73)	487.57 (61.80)	483.91 (55.48)	0.026
Blood pump flow (mL/min)	329.53 (43.31)	349.73 (52.23)	339.87 (49.11)	< 0.001
Ultrafiltration (L)	1.83 (0.97)	1.91 (0.91)	1.87 (0.94)	0.170
Vascular access				
Catheter	118 (22.14%)	75 (13.42%)	193 (17.67%)	< 0.001
AVF	415 (77.86%)	484 (86.58%)	899 (82.33%)	
Dialysis vintage (years)	5.59 (5.51)	5.84 (5.41)	5.72 (5.46)	0.450

Abbreviations: AVF, arteriovenous fistula; HDx, expanded hemodialysis; HF‐HD, high‐flux hemodialysis.

**FIGURE 2:**
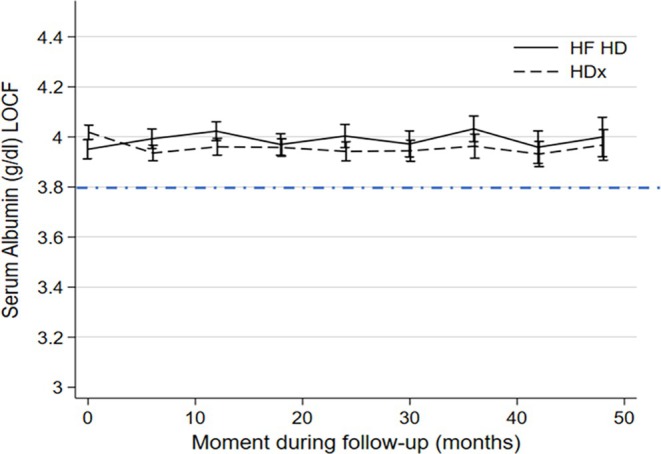
Time trends in serum albumin (mean ± SD) over 48 months according to dialyzer membrane in patients receiving HDx enabled by Theranova or high‐flux hemodialysis (HF‐HD). A serum albumin concentration of 3.8 g/dL is shown for comparison.

Serum albumin levels in the two cohorts were evaluated over time (Figure 2). No significant differences were found when comparing HDx versus HF‐HD cohorts, *p*‐value = 0.789. In addition, the percentage change from the previous measurement was estimated. When assessing all measurement times, the maximum level of change was −2.06% in HDx versus −1.82% in HF‐HD; however, at the 18‐month follow‐up, a statistically significant difference was observed in the % change between the two groups, HF‐HD −1.32% versus HDx −0.07% (*p* = 0.024), a difference that seems clinically not important. At all other measurement moments, no statistically significant differences were observed. Details are presented in Table 2.

**TABLE 2:** Serum albumin over time and percent change from previous measurements in the two cohorts.

HF‐HD				HDx			
Months	*N*	Albumin (g/dL)	Change from previous (%)	*N*	Albumin (g/dL)	Change from previous (%)	*p*
0	533	3.95	—	559	4.02		
6	482	3.99	1.07	543	3.94	−2.06	0.068
12	452	4.02	0.76	509	3.96	0.63	0.808
18	388	3.97	−1.32	456	3.96	−0.07	0.024
24	358	4.00	0.85	426	3.94	−0.39	0.406
30	320	3.97	−0.79	395	3.94	0.06	0.118
36	259	4.03	1.52	310	3.96	0.46	0.193
42	207	3.96	−1.82	278	3.93	−0.79	0.309
48	128	4.00	1.02	229	3.97	0.92	0.926

Abbreviations: HDx, expanded hemodialysis; HF‐HD, high‐flux hemodialysis.

A linear panel regression was performed to assess the association between serum albumin levels and HDx using the Theranova dialyzer while controlling for clinical and demographic confounding variables. The analysis showed no significant association between albumin levels and Theranova dialyzer use (*p* = 0.208). However, serum albumin was linked to both inflammatory and nutritional markers, including C‐reactive protein, ratio of platelets to lymphocytes, and PEW (Table 3). No multicollinearity was observed (the maximum value of VIF in the variables of the model was 1.3).

**TABLE 3:** Linear panel regression analysis for serum albumin levels.

	Coefficient	*p*	[95% confidence interval]	
Theranova dialyzer	−0.022	0.208	−0.056	0.012
C‐reactive protein (mg/L)	−0.005	< 0.001	−0.007	−0.002
Ratio platelets/lymphocytes	−0.001	< 0.001	−0.001	0.000
Protein energy wasting: yes	−0.058	< 0.001	−0.071	−0.046
Age, years	−0.006	< 0.001	−0.007	−0.004
History of diabetes: yes	−0.023	0.235	−0.061	0.015
History of major cardiovascular events: Yes	−0.020	0.349	−0.063	0.022
Urinary output ≥ 150 mL/day	−0.006	0.783	−0.046	0.035
Vascular access: arteriovenous fistula	0.071	0.004	0.023	0.120
Dialysis vintage (years)	−0.003	0.057	−0.007	0.000
Number of hospitalizations	−0.015	< 0.001	−0.023	−0.007

**FIGURE 3:**
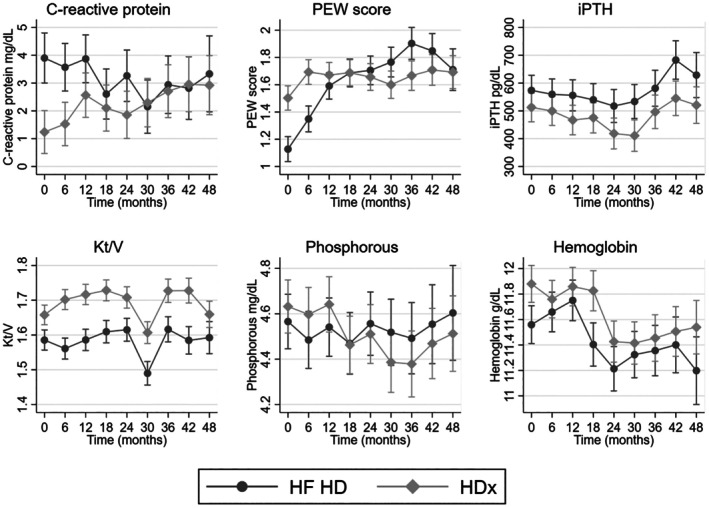
Time trends of serum biomarkers of inflammation, nutrition, and dialysis efficacy over 48 months according to dialyzer membrane in patients receiving HDx enabled by Theranova or high‐flux hemodialysis (HF‐HD). Kt/V, fractional body fluid volume cleared of urea during dialysis session; PEW, protein energy wasting; iPTH, intact parathyroid hormone.

Regarding changes over time in various biomarkers, we observed a trend toward increased inflammation (hs‐CRP) and improved nutritional status (PEW score) in the HDx group compared to the HF‐HD group, although at the end of the study period, these differences were not statistically significant (*p* > 0.05). Additionally, the HDx group showed significantly higher levels of Kt/V (*p* < 0.01) and hemoglobin (*p* = 0.02), less abnormal parathyroid hormone, that is, lower levels (*p* = 0.03), while phosphorus levels (*p* = 0.96) did not differ much between the two groups. Details in Figure 3.

## 5 Discussion

In this study, two cohorts of prevalent HD patients, one treated with expanded hemodialysis therapy enabled by the Theranova dialyzer and the other receiving conventional HD with a high‐flux dialyzer (HF‐HD), were followed up for up to 4 years to determine if there was an association of dialyzer membrane use with variations in serum albumin levels over time. The study showed as its main finding that the use of HDx therapy enabled by the Theranova dialyzer was not associated with any significant changes in serum albumin concentrations over time, and there was no significant difference as compared to the HF‐HD cohort. Furthermore, mean values of serum albumin in the two cohorts remained above 3.8 g/dL, a value that may be considered to represent the minimum normal threshold concentration of this nutritional and inflammation marker.

These results are in line with previous results reported by our group [10, 11], and with recent evidence that expanded hemodialysis enabled by Theranova dialyzer was not associated with a decrease in serum albumin levels after 6‐month follow‐up [13]. In the current study, mean serum albumin levels did not change much during long‐term follow‐up; for example, changes from baseline to follow‐up at 48 months were in HDx from 4.02 to 3.97 g/dL and in HF‐HD from 3.95 to 4.00 g/dL (Table 2). This pattern of stability in serum albumin levels in both cohorts, and without relation to the type of dialyzer used, is reassuring, and does not agree with previous reports that in chronic hemodialysis patients there is a tendency toward a slow decrease in serum albumin levels during long‐term follow‐up [14]. While the reason for this difference is not known, one may speculate that it might imply that our patients, who on average had been on preceding dialysis treatment for more than 5 years, were clinically stable. Furthermore, given the characteristics of MCO membranes, and particularly the diameter of their pores, it has been suggested that the positive effect of expanding the clearance possibilities of large uremic toxins with these membranes is at the expense of increased loss of albumin [12]. Our study shows that this is not the case, and that even after a 4‐year follow‐up, a consistent pattern of stability in albumin levels is observed, and these levels were not associated with the type of dialyzer membrane used. In parallel, our study shows that, rather than being dependent on dialyzer membrane, serum albumin levels in repeated measurements during follow‐up are associated with other factors including markers of inflammation, the type of vascular access, the diagnosis of PEW syndrome, and the presence of hospitalization events. Specifically, the link between hypoalbuminemia, inflammation, and malnutrition in the dialysis population has already been well described and highlights how serum albumin is a marker that is mainly influenced by factors such as nutritional status, age, fluid overload, inflammation, dialysis vintage, type of vascular access, losses from the dialyzer, among others [15, 16]. Likewise, while HDx has been shown to result in slightly higher albumin losses compared to HD‐HF, the variability observed is similar to that seen in OL‐HDF. Notably, clinical hypoalbuminemia or a deterioration in nutritional status over time has not been reported in any cases [17, 18].

The present study has the strength of including a substantial number of patients belonging to renal clinics that share the same clinical routines for processes and procedures. In addition, monitoring for up to 4 years allows us to observe trends that shorter‐term monitoring cannot capture. Longitudinal follow‐up designs with repeated measurements over time are not only informative about trends, but they may also improve the capacity of some variables to predict health outcomes [19].

As weaknesses of the present study, it should be noted that since it is an observational study, there is always the risk of confounding, that additional variables, measured or not measured, may confuse the eventual association between the exposure variable (in this case the type of membrane) and the outcome (in this case albumin levels in repeated measurements during follow‐up). However, the panel linear regression model employed effectively controls potential confounders. It is important to note that, as this is a non‐randomized study, there remains the possibility of indication bias, which could lead to imbalances in both observed and unobserved variables. Consequently, the results should be interpreted with this limitation in mind. Additionally, we lack data to assess albumin leakage, an aspect about which some have warned [20], in the sense that MCO dialyzers could facilitate greater loss of albumin to the dialysate.

## 6 Conclusion

Time‐varying serum albumin levels did not associate with the type of dialyzer in this cohort study of patients treated by either HDx therapy enabled by Theranova dialyzer or by conventional maintenance hemodialysis using high‐flux membrane and followed for up to 48 months. Instead, PEW, inflammation, high age, vascular access other than arteriovenous fistula, and hospitalization events were associated with variations in serum albumin concentrations over time.

## Ethics Statement

The study protocol was approved by the Clinical Research Ethics Committee of the Cardioinfantil Foundation (May 31, 2023, minutes, item number 019), which exempted the study from the requirement of informed consent since the study does not contain identifiable information and is a retrospective observational study.

## Conflicts of Interest

Dr. Castillo is an employee of Renal Care Services Soacha, Ms. Vesga is an employee of Renal Care Services Colombia, Dr. Sanabria is an employee of Renal Care Services‐Latin America, Dr. Rivera and Dr. Rutherford are employees of Baxter Healthcare Corporation. Dr. Sanchez is an employee of the Faculty of Medicine at the National University of Colombia and has received an honorarium for statistical analysis from Baxter Healthcare Corporation. Dr. Oliveros is an employee of the Faculty of Medicine at Sabana University and has received an honorarium for statistical analysis from Baxter Healthcare Corporation. Dr. Lindholm is an employee of Karolinska Institutet and a former employee of Baxter Healthcare Corporation and has received a research grant from Baxter Healthcare Corporation to Karolinska Institutet.

## Data Availability

The entire protocol and database for the study are available from the principal investigator (juan_castillo@baxter.com).
